# Home Physical Exercise Interventions in Chronic Non-Specific Low Back Pain: Systematic Review and Multivariate Meta-Analysis

**DOI:** 10.3390/healthcare13172094

**Published:** 2025-08-22

**Authors:** Diego Lapuente-Hernández, Marina Gil-Calvo, Juan Nicolás Cuenca-Zaldívar, Alberto Carcasona-Otal, Pablo Herrero, Ángel Matute-Llorente

**Affiliations:** 1 Department of Physiatry and Nursing, Faculty of Health Sciences, University of Zaragoza, 50009 Zaragoza, Spain; d.lapuente@unizar.es (D.L.-H.); pherrero@unizar.es (P.H.); 2 iHealthy Research Group, Instituto de Investigación Sanitaria (IIS) Aragon, HCU Lozano Blesa, 50009 Zaragoza, Spain; acarcasona@unizar.es; 3 Faculty of Physical Activity and Sports Sciences, AMREDyS, Universidad de León, 24401 León, Spain; 4 Grupo de Investigación en Fisioterapia y Dolor, Departamento de Enfermería y Fisioterapia, Facultad de Medicina y Ciencias de la Salud, Universidad de Alcalá, 28801 Alcalá de Henares, Spain; nicolas.cuenca@salud.madrid.org; 5 Research Group in Nursing and Health Care, Puerta de Hierro Health Research Institute-Segovia de Arana (IDIPHISA), 28222 Majadahonda, Spain; 6 Primary Health Center “El Abajón”, 28231 Las Rozas de Madrid, Spain; 7 Department of Physiatry and Nursing, Faculty of Health and Sport Sciences, EXER-GENUD, University of Zaragoza, 22001 Huesca, Spain; amatute@unizar.es

**Keywords:** exercise therapy, functional disability, low back pain, pain intensity, multivariate meta-analysis

## Abstract

**Background/Objectives**: Low back pain is considered one of the leading causes of disability. Up to 90% of cases are classified as non-specific, which, if prolonged for at least 12 weeks, is considered non-specific chronic low back pain (NSCLBP). Physical exercise is one of the selected treatments for NSCLBP. Interest in the use of remote interventions has recently emerged. The main objective was to analyze the effect of home exercise interventions to reduce pain intensity and functional disability in individuals with NSCLBP. **Methods**: A systematic review was conducted in April 2024. Both multivariate and univariate meta-analysis was performed with the difference before and after treatment, adjusting both models with a meta-regression that included the covariates age and body mass index (BMI). Heterogeneity was analyzed with Cochran’s Q test as well as with the I^2^ estimator, and effect size was calculated with Hedges’G. **Results**: A total of six studies, with moderate–high methodological quality and a heterogeneous risk of bias, were included. There was a statistically significant pre-/post-treatment effect on functional disability (moderate effect: Hedge’s g = 0.69, *p* = 0.018) and pain intensity (large effect: Hedge’s g = 1.11, *p* = 0.007) in both univariate and multivariate (moderate effect: Hedge’s g = 0.77) meta-analyses when comparing unsupervised home exercise with supervised in-person exercise, in favor of the latter. This effect was significantly moderated by BMI (*p* = 0.003 for both outcomes) negatively. **Conclusions**: Unsupervised home exercise appears to be less effective than supervised in-person exercise in effectively reducing pain intensity and functional disability in the short term in individuals with NSCLBP.

## 1. Introduction

One of the most frequent reasons for seeking medical care is pain. The “Global Burden of Disease Study 2021” has highlighted that low back pain (LBP) is the leading cause of disability-adjusted life years globally [1]. This health condition is responsible for the highest global burden of disease, generating an impact not only on personal lifestyle but also on healthcare systems [2,3]. Indeed, there is a growing number of public initiatives focused on the treatment and prevention of low back pain [4].

In the context of LBP, most cases (85–90%) fall under the category of non-specific LBP. When the duration of non-specific LBP reaches at least 12 weeks, it is considered non-specific chronic low back pain (NSCLBP). Many factors may influence NSCLBP, and it stems from a complicated interplay between physical, psychological, social, professional, and even genetic factors [5,6,7].

It is generally recommended that individuals with NSCLBP stay active, as it has been demonstrated that prolonged periods of inactivity can have a negative impact on the recovery of functionality [8,9]. Almost all international clinical guidelines recommend physical exercise as a basic treatment for individuals with NSCLBP, as well as being one of the best non-pharmacological approaches to reduce pain and functional disability in both the short and long term [10,11]. At present, there is no conclusive evidence regarding which form of exercise provides the most benefits, whether it is aerobic, resistance, or flexibility training. However, there is evidence demonstrating the effectiveness of exercise compared to no treatment, placebo, and conventional interventions such as manual therapy or usual treatment from general practitioners [9].

When dealing with NSCLBP, a multidisciplinary and biopsychosocial approach must be considered due to its complex nature [11]. Additionally, treating long-standing conditions like NSCLBP requires prolonged follow-up periods. For this reason, treatment barriers such as a lack of commitment or time and high financial costs appear [12]. Therefore, treatment modalities that are easily accessible, cost-effective, and that reduce the patients’ effort are required. This has led to an increase in remote and digital interventions to support treatments [12]. Telerehabilitation can be used to monitor compliance and track results, while also providing positive feedback to patients [9,13]. This methodology is increasing rapidly and has proven to be a cost-effective alternative to traditional methods in the population with chronic musculoskeletal pain [14].

Most treatment approaches for NSCLBP emphasize the incorporation of home-based exercise alongside supervised in-person sessions; hence, various reviews have evaluated the effect of home exercise programs in managing NSCLBP [15,16,17]. However, the observed outcomes cannot be exclusively attributed to physical exercise, as many of the included studies in these reviews combined exercise with other therapeutic interventions. Furthermore, several studies included participants with both acute and chronic non-specific LBP, adding heterogeneity to the results.

Therefore, the main objective of this study was to analyze the effectiveness of home physical exercise interventions in isolation in reducing pain intensity and functional disability in individuals with NSCLBP. The following were proposed as specific objectives for this same population: (1) to compare the effectiveness between home and supervised in-person physical exercise interventions; (2) to compare the effectiveness between unsupervised and supervised via video home physical exercise interventions; and (3) to compare the effectiveness between home physical exercise interventions and no intervention.

## 2. Materials and Methods

This systematic review and meta-analysis were conducted according to the Preferred Reporting Items for Systematic Reviews and Meta-Analyses (PRISMA) standard protocol [18]. This study has been registered with the PROSPERO international prospective register of systematic reviews (reference number CRD42023437258).

### 2.1. Eligibility Criteria

To define the research question and, therefore, determine the inclusion and exclusion criteria when selecting the different studies, the PICOS formula was used [18].

- Population: Adult population (>18 years), experiencing NSCLBP, of three months or more of evolution. Therefore, all types of traumatic LBP and those due to specific pathologies (spondylolisthesis, stenosis, infectious and/or inflammatory processes, tumors, neurological conditions, etc.) were excluded.
- Intervention: Prescription of physical exercise exclusively at home/online (unsupervised or supervised via video). This intervention could include and even combine different modalities of physical exercise, but it could not include or be compared with other therapeutic approaches.
- Comparator/control: The comparator group must adhere to the same physical exercise prescription as the intervention group, but with supervision provided exclusively through face-to-face/in-person sessions. This approach ensures that any observed differences between groups can be attributed solely to the mode of exercise supervision (supervised versus unsupervised), rather than variations in the exercise program or type of exercise performed. Additionally, the control group may include participants receiving general advice or counseling on physical activity, exercise, and ergonomics or those maintaining their usual activities of daily living without any specific intervention.
- Outcomes: Pain intensity and functional disability. No particular scale or questionnaire was designated to quantify these outcomes.
- Study design: Only randomized clinical trials (RCTs) published in English or Spanish. Therefore, any other type of clinical trial was excluded. Reviews or meta-analyses, book sections, conference abstracts, and protocols were also excluded.

### 2.2. Data Sources and Search

A bibliographic search was conducted on 23 December 2024, using PubMed, Web of Science, Scopus, and SportDiscus databases. The search strategy was adapted to the terms and indexing functions of each of the databases used. Regarding the search terms, two categories were defined: (1) study population (“Chronic low back pain”, “CLBP”, “Nonspecific chronic low back pain”, “Non-specific chronic low back pain”, “NSCLBP “, “NSLBP”, “Chronic nonspecific low back pain”, “Chronic non-specific low back pain”, “Chronic, nonspecific low back pain”, and “Chronic, non-specific low back pain”); (2) type and modality of intervention (“Exercise*”, “Physical activity*”, “Physical exercise”, “Exercise training”, and “Exercise therapy”) + (“Home environment”, “Home”, “Domiciliary”, “ Remote”, “Video”, “Virtual”, “Telerehabilitation”, “Telemedicine”, “Mobile Health”, “Telehealth”, “mHealth”, and “eHealth”). These terms were chosen after conducting an initial search of the literature and identifying various relevant keywords. The complete search strategies used together with the filters applied in each database are shown in detail in Table S1.

### 2.3. Study Selection

To assess if the studies fulfilled the inclusion criteria, two investigators (D.L.-H. and A.C.-O.) independently evaluated each report. They followed a standardized methodology after agreeing on the search equations. Initially, all records from the four databases were gathered and imported into the “Mendeley version 2.98.0” reference manager to identify and remove potential duplicates. Subsequently, an initial screening based on title and abstract was performed to select relevant studies. The next phase involved a comprehensive review of the full texts of the selected studies to determine their eligibility based on predefined inclusion and exclusion criteria. Any discrepancies in the selection process were resolved through consultation with a third researcher (M.G.-C.) to achieve consensus.

### 2.4. Data Extraction Process

Two reviewers (D.L.-H. and A.C.-O.) conducted independent data collection from the studies and subsequently cross-referenced their findings for consistency. Any discrepancies were resolved through consultation with a third party (M.G.-C.).

From each included study, the following details were extracted: references (author and date); sample characteristics (for each group: size, sex, mean age, mean body mass index, and mean pain duration); intervention; comparator/control; outcomes of interest (related to pain intensity and functional disability); follow-up/assessment moments; and main results. Moreover, specific details regarding home interventions were gathered from each study, encompassing the following: type of intervention; weekly frequency and duration of sessions; session volume (number of sets and repetitions); home follow-up form; aids, tools, and any additional aspects.

### 2.5. Assessment of Methodological Quality and Risk of Bias

The procedure for assessing both the methodological quality and risk of bias of the included studies was the same. First, the two reviewers were trained in the use of the scales to be used to standardize and optimize this process. Both reviewers (D.L.-H. and A.C.-O.) worked independently, and the results were then compared. The intervention of a third reviewer was not required at any time.

The methodological rigor of the studies included in the analysis was evaluated using two distinct tools tailored to different aspects of study quality. Firstly, the PEDro scale, explicitly designed for RCTs, was employed. Comprising 11 criteria, each study was scored based on whether it met these criteria, with a score of one assigned for compliance and zero for non-compliance. The criteria encompassed aspects such as the establishment of eligibility criteria, study design, and result interpretability. Notably, the maximum achievable score was 10, as the first criterion was not factored into the final assessment. Scores above 6 indicated a classification of “high quality”, while those between 4 and 5 were deemed “moderate quality”, and scores below 4 were categorized as “low quality” [19,20].

Additionally, the risk of bias within the included studies, all of which were RCTs or studies employing random assignment, was evaluated using the Cochrane Risk of Bias 2.0 (RoB2) tool [21]. This tool assessed bias across five domains: the randomization process, deviations from planned interventions, missing outcome data, outcome measurement, and selection of reported outcomes. Each domain was evaluated for the level of risk, categorized as “low risk”, “some concern”, or “high risk” of bias.

### 2.6. Statistical Analysis

The statistical analysis was conducted using the program R Ver. 4.1.3 (R Foundation for Statistical Computing, Institute for Statistics and Mathematics, Welthandelsplatz 1, 1020 Vienna, Austria).

Means and standard deviations were requested from the corresponding authors by email if the articles did not include this information. If a response could not be obtained, articles that displayed results using median and interquartile ranges were converted to mean and standard deviation using the appropriate formulas [22,23], in which the intervals of confidence were provided instead of the standard deviation [24]. Finally, in the articles that did not report any data, this information was extracted from the graphs using the WebPlotDigitizer Ver. 4.6 program [25].

A meta-analysis was performed on the pre- and post-treatment differences. This was calculated with the appropriate formulas when the studies did not report it [24]. To be conservative, a pre-/post-treatment correlation coefficient of 0.7 was assigned [26], as has been completed in other studies [27,28,29,30,31]. The group that performed unsupervised home physical exercise was selected as the intervention group, while the groups that performed supervised physical exercise in person were selected as the control group. In a study by Karaduma and Ataş Balci [32], data from the tele-supervised home physical exercise group were not included in the quantitative analysis; they were only considered for the qualitative analysis, as it was the only study that compared unsupervised and supervised home physical exercise.

A multivariate meta-analysis (MMA) was applied with the standardized mean difference (SMD) between the pain intensity and functional disability scales using correlations between both types of scales already described in the literature [33,34]. Univariate meta-analyses were also performed for pain intensity and functional disability using the mean difference (MD) as the effect size in pain intensity when all studies used the same scale and the SMD in functional disability when studies used different scales. The models, in both cases, were adjusted using meta-regression, incorporating age and body mass index (BMI) as covariates. These variables were selected due to their consistent availability across most studies and their established influence on exercise outcomes [35,36].

In the MMA, the likelihood ratio test (LRT) and the lowest value in the Akaike information coefficient (AIC) were used to select the fixed or random effects model. In contrast, in the univariate analysis, a random effects model was applied, given the heterogeneity between the studies. Heterogeneity was analyzed by estimating the between-study variance τ^2^, with Cochran’s Q test as well as with the I^2^ estimator, defining heterogeneity as not important (<30%), moderate (30–50%), large (50–75%), and important (>75%). The effect size was calculated with Hedges’ G, defining it as small (<0.2), moderate (0.2–0.8), and large (>0.8). Publication bias was analyzed with the Egger’s test. The forest, caterpillar, and funnel plots were created following the recommendations of Fernández-Castilla et al. [37] for multivariate meta-analyses.

## 3. Results

### 3.1. Study Selection

**Figure 1:**
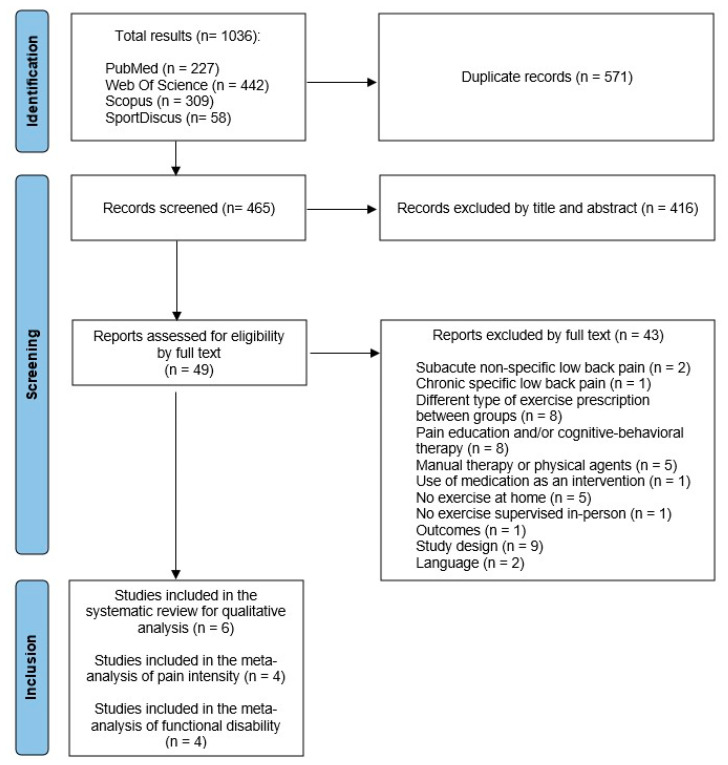
PRISMA flow diagram from search strategy.

The bibliographic search identified a total of 1036 studies. After applying the inclusion criteria, six studies were included in the systematic review, of which four were included in the meta-analysis for quantitative analysis. The flow diagram (Figure 1) shows in more detail the study search and selection process, together with the different reasons for exclusion throughout the process, following the PRISMA criteria [18].

### 3.2. Study Characteristics

Table 1 summarizes the main characteristics of the six included studies, grouped in alphabetical order according to the last name of the first author.

#### 3.2.1. Sample Size and Characteristics

The sample size varied between 48 and 64 subjects, with a total of 351 individuals among all groups. The total review sample consisted of 57.5% women (n = 202) and 42.5% men (n = 149). The mean age of the sample was 51.2 ± 9.0 years. Body mass index (BMI) was reported in all studies, except by Matarán-Peñarrocha et al. [41], with a mean of 27.3 ± 5.8 kg/m^2^. The mean duration of pain was reported in only two studies [32,41], with the mean of these samples being 115.3 ± 20.1 weeks.

#### 3.2.2. Design of the Groups and Description of the Intervention

In 66.7% of the studies, an unsupervised home physical exercise group was compared to a supervised in-person physical exercise group [32,38,39,41]. In the comparison, in the remaining 33.3%, the unsupervised home physical exercise group was compared to no intervention, i.e., continuing with their usual activities of daily living [40,42]. In addition, the study by Karaduman and Ataş Balci [32] included a group performing tele-supervised home physical exercise by a physiotherapist. The specifications of the home interventions of each of the included studies are explained in detail in Table 2.

The duration of the interventions ranged from 4 to 12 weeks, with 4 and 8 weeks being the most used (each in 33.3% of the studies) [32,39,41,42]. The number of sessions per week ranged from 2 to 7 days per week, with 3 sessions being the most commonly used [32,41,42]. The duration of each session was between 20 and 60 min, leaving 5–10 min for warm-up and cool-down. About the volume of the sessions, the most frequent recommendation was to perform 3 series, varying between 1 and 4, with 10 to 20 repetitions for each exercise [32,38,39,40,41] and 20 to 30 s for each stretch [39]. In none of the studies was there any mention of the parameter intensity of the exercise program.

All home physical exercise interventions were based on core exercises, either resistance, stabilization, and/or mobility, except in the study by Zadro et al. [42], which included full-body resistance in addition to aerobic exercise. Moreover, some of these interventions included lower-limb exercises in addition to core [39,40,41].

#### 3.2.3. Outcomes and Follow-Up

All the studies included analyzed the two outcomes of interest (pain intensity and functional disability), at least before and after the intervention.

On the one hand, pain intensity was evaluated with three scales. The most frequent was the 0–10 visual analogue scale (VAS) since it was used in 50.0% of the studies [38,39,41]. The 0–10 numeric rating scale (NRS) was used in two studies [39,42] and the 0–11 Borg scale in one study [40].

On the other hand, functional disability was evaluated with three questionnaires and one functional test. The most used questionnaires were the 0–100 Oswestry disability index (ODI), used in 66.7% of studies [32,39,40,41], and the 0–24 Roland–Morris disability questionnaire (RMDQ), used in 50.0% of studies [38,41,42]. The patient-specific functional scale (PSFS) was used only in the study by Zadro et al. [42]. Finally, about the functional tests, the five-times sit-to-stand test (FTSTS) was used.

Regarding the follow-up moments, in 50.0% of the included studies, additional follow-up was carried out, ranging from 12 weeks to 5 years [39,40,41].

### 3.3. Methodological Quality and Risk of Bias

The methodological quality, as assessed by the PEDro scale, is summarized in Table 3. Four out of six studies (66.7%) obtained a score of six or more points [32,38,41,42], which is considered high quality, while the remaining 33.3% obtained five points and, therefore, were established as moderate quality [39,40]. The average score of the studies was 6.3 out of 10, with a range between 5 and 8. Blinding of the subjects or the therapists was not performed in any of the studies, a common practice in clinical trials involving physical exercise as an intervention.

**Figure 2:**
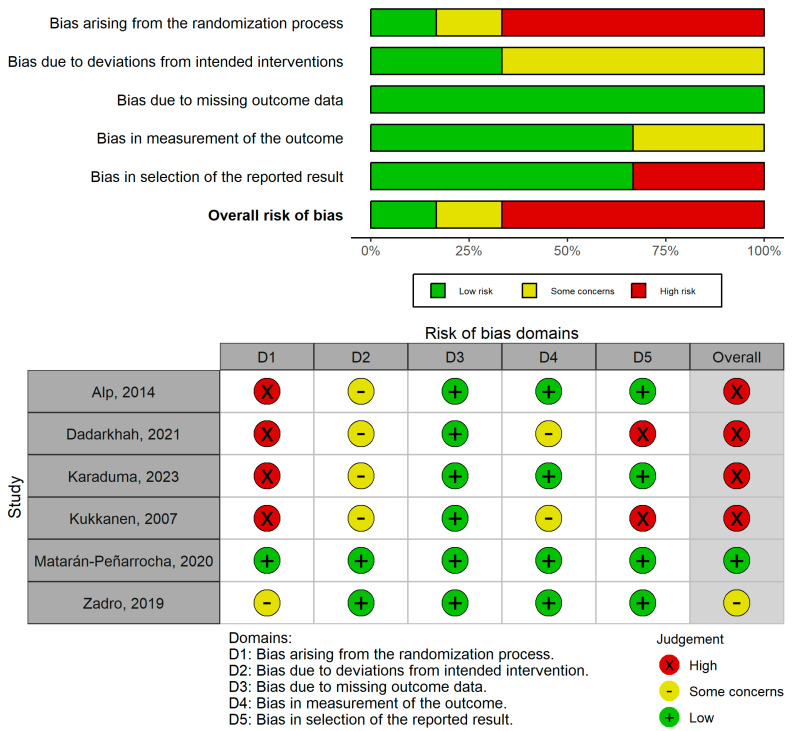
RoB2 risk of bias assessment plots [32,38,39,40,41,42].

The risk of bias of the included studies measured with the ROB2 tool is summarized in Figure 2. One out of six studies (16.7%) was considered low risk [41], another 16.7% with some concerns [42], and the remaining 66.7% was considered high risk [32,38,39,40].

### 3.4. Review Results

#### 3.4.1. Unsupervised Home Physical Exercise vs. Supervised In-Person Physical Exercise

The results observed for both pain intensity and functional disability were similar when comparing unsupervised home physical exercise and supervised in-person physical exercise. In the studies by Alp et al. [38] and Dardarkhah et al. [39], no differences were found between the two interventions, while in the studies by Karaduman and Ataş Balci [32] and Matarán-Peñarrocha et al. [41], statistically significant differences in favor of supervised in-person physical exercise were found for both variables immediately post-intervention, but without clear results at longer follow-up.

#### 3.4.2. Unsupervised Home Physical Exercise vs. No Intervention

In this case, the results between variables were somewhat different. Statistically significant differences in favor of unsupervised physical exercise were observed for the improvement of pain intensity in the studies of Kuukkanen et al. [40] and Zadro et al. [42], while for functional disability, no differences were found in these same studies.

#### 3.4.3. Unsupervised Home Physical Exercise vs. Tele-Supervised Home Physical Exercise

Although this analysis was only carried out in the study by Karaduman and Ataş Balci [32], the results showed statistically significant differences in favor of tele-supervised home physical exercise in reducing pain intensity. At the same time, there was no difference in functional disability.

### 3.5. Meta-Analysis Results

The ANOVA test was significant (X^2^(3) = 9.37, *p* = 0.025), with a lower AIC in the random effects model (11.61 vs. 14.98), indicating that the fit of the full random effects model was better than the reduced fixed effects model.

In the MMA, Cochran’s Q test was significant (X^2^(6) = 23.25, *p* = 0.001), indicating the presence of heterogeneity. The inclusion of moderators reduced the level of heterogeneity both in functional disability with an I^2^ that ranged from a large value of 68.39% (*τ*^2^ = 0) to a large one of 56.88% with age and practically zero for 2.49% with BMI, as in pain intensity, with an I^2^ from a large value of 80.71% (*τ*^2^ = 0.357) to a large one of 72.93% with age and 72.26% with BMI. The presence of a positive and low correlation between both variables was verified (*ρ* = 0.122). There was a significant pre-/post-treatment effect on functional disability (*p* = 0.001) and pain intensity (*p* = 0.001), which were also significantly moderated by BMI (*p* = 0.003 and *p* = 0.003, respectively) negatively; i.e., the higher the BMI, the lower the scores on the functional disability and pain intensity scales, and therefore, the greater reduction in both. The percentage of variance explained by the model was large in functional disability (R^2^ = 1) and null in pain intensity (R^2^ = 0) (Table 4 and Figure S1).

**Figure 3:**
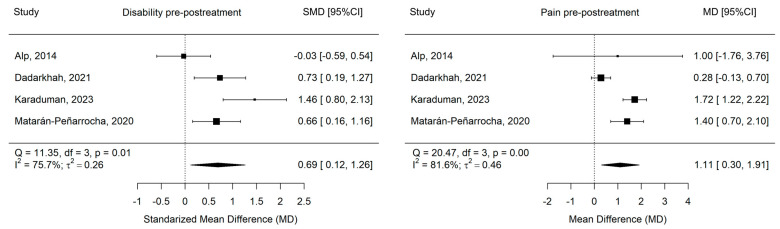
Forest plots by outcome; 95% CI: 95% confidence interval [32,38,39,41].

The univariate meta-analysis showed a moderate and significant effect on functional disability (Hedge’s g = 0.69 [0.12, 1.26], Z = 2.36, *p* = 0.018) and large and significant effect on pain intensity (Hedge’s g = 1.11 [0.30, 1.91], Z = 2.70, *p* = 0.007), with a greater reduction in functional disability and pain intensity in the supervised in-person physical exercise group (control group). In both cases, heterogeneity was important (Figure 3). The meta-regression showed that neither age nor BMI had a significant effect on functional disability or pain intensity (X^2^(1) = 2.66, *p* = 0.103 and X^2^(1) = 0.14, *p* = 0.706, respectively).

**Figure 4:**
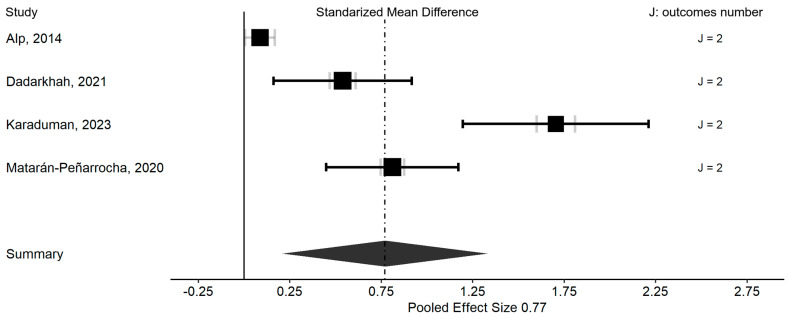
Multivariate forest plot with sample size and effect by study. In terms of precision measures, black symbols represent the combined outcome’s effect size, while gray symbols indicate the averaged effect size due to each separate outcome, with the width proportional to the averaged outcomes’ variance and the thickness to the number of outcomes [32,38,39,41].

The multivariate forest plot shows how the combined effect of all studies was moderate and significant (Hedge’s g = 0.77), with a reduction in functional disability and pain intensity in the supervised in-person physical exercise group compared to the unsupervised home physical exercise (intervention group). It was observed that the total precision of the studies was greater than the precision derived from the sample size, indicating low variability between studies (Figure 4). The caterpillar plots of both the measurements (Figure S2A) and the studies (Figure S2B) show the significance of the effect due to a greater number of both measurements and studies with significant results.

Egger’s test was non-significant (t(5) = −0.77, *p* = 0.475), indicating the absence of heterogeneity. Both the funnel plot with the measurements (Figure S3A) and with the studies (Figure S3B) show the effects distributed symmetrically around the central axis and within the limits of significance, which corroborates the absence of publication bias.

## 4. Discussion

The primary objective of this systematic review and meta-analysis was to analyze the effectiveness of home physical exercise interventions in reducing pain intensity and functional disability among individuals with NSCLBP. This evaluation was based on an analysis of six studies with a moderate to high level of methodological quality, albeit with some risk of bias. Specifically, four of the six studies included (66.67%) were rated as having a high risk of bias, which significantly limits the reliability of the observed effects and must be considered when interpreting the results. Initially, a qualitative analysis was carried out to compare the effects of unsupervised home physical exercise vs. supervised in-person physical exercise, vs. no intervention, and vs. tele-supervised home physical exercise. Subsequently, with four of these studies, a quantitative analysis was carried out using univariate and multivariate meta-analysis to specifically compare the effect of unsupervised home physical exercise vs. supervised in-person physical exercise.

The meta-analysis results demonstrated that unsupervised home physical exercise is not as effective as supervised in-person exercise in reducing pain intensity and functional disability in NSCLBP. These differences were statistically significant, with a large effect size for pain intensity and moderate for functional disability and the combined outcomes. These findings are consistent with a previous systematic review by Hayden Van Tulder and Tomlinson [43], which indicated that personalized exercise programs, including supervised resistance and/or mobility exercises, improve pain and functionality in individuals with NSCLBP.

Although the quantitative analysis focused on short-term and pre- and immediate post-treatment differences due to the lack of long-term data, the results from a systematic review suggest a trend toward equivalence in the effect of supervised in-person exercise and unsupervised home exercise over more extended follow-up periods, similar to those found in the meta-analysis of Hayden et al. [9]. Therefore, home exercise is recommended for NSCLBP in the medium to long term due to its cost-effectiveness and ease of participation, provided adequate professional follow-up and feedback through phone calls or session diaries [44] or even face-to-face sessions are ensured [45].

The multivariate analysis indicated that BMI significantly influences the combined improvement of pain intensity and functional disability in individuals with NSCLBP. Since the direction in which it influences is negative, individuals with a higher BMI may benefit more from supervised in-person exercise by a healthcare professional. This is consistent with BMI being an important risk factor associated with the progression and prognosis of NSCLBP [46,47], so exercise, partly aimed at attempting to modify BMI and what that entails for the individual, may reduce the disease burden [48]. There are also studies that show that BMI does not predict changes in pain intensity or functional disability in the population with NSCLBP [49], casting doubt on the validity of BMI as a single measurement, as it does not allow differentiation between muscle mass and fat mass, the latter playing a greater role in the pathogenesis of LBP [50]. Age, however, did not emerge as a significant moderator, indicating that supervised in-person exercise benefits individuals with NSCLBP regardless of age.

From the results of the systematic review, when comparing unsupervised home physical exercise with no intervention, the expected results were not found, since statistically significant differences were only found in favor of exercise in the improvement of pain intensity, but not in functional disability. This may be partly because the effect of active movement/exercise-based treatment in chronic pain may be 30% due to the placebo effect and 10% due to spontaneous improvement, the natural history of the condition, and regression to the mean [51]; hence, possibly the non-exercise population improved simply by the passage of time. Thus, home exercise alone may not be the optimal strategy for NSCLBP; additional therapeutic strategies, such as pain education, should be prioritized [52]. These strategies should address the nature of pain, its subjective and multidimensional aspects, and the importance of self-management and active coping mechanisms [53]. Furthermore, this can be directly related to the results discussed above, since as the follow-up time of individuals with NSCLBP increases, the likelihood that the effects of the intervention are equalized is greater, regardless of the exercise modality used.

The systematic review also found differences in favor of tele-supervised home physical exercise over unsupervised exercise, particularly for pain intensity. This result may highlight the importance of aspects such as communication, confidence, and comprehensive care and the creation of positive experiences [54,55,56]. It may also be because performing the exercises simultaneously with viewing the professional on the screen leads to a more efficient learning capacity and optimal performance [57]. The development of “telehealth” interventions, which include online platforms, mobile applications, and video calls, has been attributed to various factors such as user demand for innovative rehabilitation methods, the globalization of health systems, and pressure to reduce health care costs [58]. The effectiveness of online platforms has already been demonstrated in pathologies such as cardiovascular diseases [59], obesity [60], chronic obstructive pulmonary disease [61], or stroke [62,63], but also in musculoskeletal conditions [64], and even in chronic pain conditions, including NSCLBP [64]. However, further research is needed to confirm their effectiveness in this condition [65,66,67].

Due to the limited number of studies, further analysis of exercise program duration, frequency, and volume was not feasible. The programs ranged from 4 to 12 weeks, with 2 to 7 sessions per week, each lasting 20 to 60 min, and comprising 10 to 20 repetitions per exercise, like what was found in a previous meta-analysis on chronic neck pain [68]. In the meta-analysis of Quentin et al. [15], in the NSCLBP population, training volume was not associated with better outcomes, but longer follow-up was associated with greater improvements. However, in this same population, it has been found that high-dose exercise programs (>20 h of exercise in interventions of at least 6 weeks) may be more effective than lower doses (<20 h) [43]. Additionally, spreading exercise sessions throughout the day might increase benefits and adherence [69].

Although intensity is one of the most relevant parameters when prescribing and evaluating the effectiveness of exercise-based interventions, none of the studies included mentioned the intensity prescribed in home exercise programs. This limits the ability to assess the dose–response relationship or compare programs based on their physiological demands. The lack of supervision in home-based programs may make it difficult to standardize or accurately report intensity. Future research should, therefore, include clearly defined intensity metrics, such as percentage of one repetition maximum, perceived exertion scales, or heart rate zones, to improve the interpretability and reproducibility of results.

Since the evidence suggests that one type of exercise is not superior to another, the choice of exercise for NSCLBP should depend on a person’s preferences and expectations, professional training, associated costs, and safety [70]. However, other factors should be considered when scheduling exercise in the population with NCSLBP, such as BMI. Initially, higher supervision levels may be necessary for individuals beginning exercise for NSCLBP, but long-term strategies should focus on promoting self-management and load management. Periodic follow-ups and providing patients with tools to manage relapses during the process can be helpful. The main objective of these programs should be to increase the individual’s ability to control their condition, reduce supervision needs, and promote an active and healthy lifestyle. To achieve this, a multicomponent approach may be required, involving educational interventions, psychological strategies, behavioral methods to enhance adherence and promote behavior change, and even social support, as suggested in models of self-management for people with long-term conditions [71].

Among the main limitations of the review, it is worth highlighting the following: (1) more than a half of the included studies present a high risk of bias according to ROB2; (2) other socio-demographic data, such as educational status, lifestyle, sleep quality, weekly physical activity, and medication use, have not been taken into account; factors that directly influence the development and evolution of the NSCLBP; (3) the study sample is made up of more than 55% women, in whom it has been seen that the pain perception and responses to treatment are different compared to men [72]; and (4) as the follow-up of the interventions was relatively short, it was only possible to carry out the meta-analysis on the pre-/post-treatment effect and not on the medium–long term. Nonetheless, the methodological quality of the studies, the introduction of a quantitative statistical analysis through the univariate and multivariate meta-analyses carried out, and the absence of heterogeneity and publication bias of the studies included in the meta-analysis enhance its robustness.

To our knowledge, this is the first systematic review and meta-analysis that includes exclusively home physical exercise interventions in the population with NSCLBP. For future research, (1) multicomponent exercise programs that combine at least muscular resistance, mobility, and aerobic exercise should be studied in this population; (2) it would be interesting to analyze if better programmed and planned interventions are more effective than standardized exercises, thus making it easier to replicate the intervention in real clinical practice; (3) studies are needed that compare exercise programs that differ exclusively in parameters such as intensity, frequency, and/or volume, and these intervention characteristics should be systematically analyzed as covariates or potential moderators to determine better their influence on clinical outcomes such as pain intensity and functional disability; and (4) studies comparing the effectiveness of unsupervised home exercise vs. supervised in-person exercise in the long term are also needed.

## 5. Conclusions

Unsupervised home physical exercise appears to be less effective in the short term in reducing pain intensity and functional disability in individuals with NSCLBP compared to supervised in-person physical exercise and less effective in reducing pain intensity compared to tele-supervised home physical exercise. Nevertheless, it demonstrates greater effectiveness in relieving pain intensity compared to no intervention. However, further research employing rigorous methodology is needed to strengthen the evidence base in this area in the NSCLBP population, as the current body of literature is characterized by a high risk of bias, which limits the reliability and generalizability of the present conclusions.

## Figures and Tables

**Table 1:** Main characteristics of the included studies.

Author and Year	Sample Characteristics	Intervention	Comparator/ Control	Outcomes	Follow-Up Moments	Main Results
Alp, 2014 [38]	IG: n = 24 (24 M); MA = 51; BMI = 30.3 ± 5.8 CG: n = 24 (24 F); MA = 48; BMI = 29.2 ± 5.4	Unsupervised core exercises at home	Supervised in-person core exercises	Pain intensity (VAS) Functional disability (RMDQ and FTST)	Baseline and 12 weeks	Intragroup SSI in VAS (IG: *p* = 0.007; CG: *p* < 0.001), RMDQ (IG: *p* = 0.005; GC: *p* = 0.001) and FTST (*p* < 0.001) No between-group SSI (VAS: *p* = 0.385; RMDQ: *p* = 0.779; FTST: *p* = 0.733)
Dadarkhah, 2021 [39]	IG: n = 28 (16F/12M); MA = 50.0 ± 8.6; BMI = 27.0 ± 2.6 CG: = 28 (16F/12M); MA = 49.0 ± 9.3; BMI = 28.0 ± 3.9	Unsupervised core exercises at home	Supervised in-person core exercises	Pain intensity (VAS) Functional disability (ODI)	Baseline, post (4 weeks), and 12 weeks	Intragroup SSI in VAS and ODI (*p* < 0.05) No between-group SSI (VAS: *p* = 0.93; ODI: *p* = 0.74)
Karaduman, 2023 [32]	IG-1: n = 22 (15F/7M); MA = 44.0 ± 17.1; BMI = 26.9 ± 4.7; PD = 21.6 ± 5.0 weeks IG-2: n = 22 (13F/9M); MA = 46.3 ± 12.3; BMI = 26.4 ± 4.2; PD = 21.3 ± 4.3 weeks CG: n = 22 (2F/20M); MA = 46.5 ± 12.7; BMI = 25.6 ± 4.0; PD = 20.7 ± 3.7 weeks	IG-1: Unsupervised core exercises at home IG-2: Tele-supervised core exercises at home	Supervised in-person core exercises	Pain intensity (NRS) Functional disability (ODI)	Baseline and post (4 weeks)	Intragroup SSI in NRS (*p* < 0.001) and ODI (*p* < 0.001) Between-group SSI in favor of the CG and IG-2 in NRS (*p* < 0.017) and in favor of the CG in ODI (*p* < 0.05)
Kuukkanen, 2007 [40]	IG: n = 29 (14F/15M); MA = 41.0 ± 8.1; BMI = 25.6 ± 13.1 CG: n = 28 (15F/13M); MA = 40.0 ± 8.9; BMI = 25.5 ± 10.5	Unsupervised core exercises at home	Usual activities of daily living	Pain intensity (Borg scale) Functional disability (ODI)	Baseline, post (12 weeks), 6 and 12 months, and 5 years	Intragroup SSI in Borg and ODI post (*p* < 0.05) Between-group SSI in favor of the IG at 5 years in Borg (*p* = 0.01), but no SSI in ODI (*p* = 0.270)
Matarán-Peñarrocha, 2020 [41]	IG: n = 32 (15F/17M); MA = 53.2 ± 8.0; PD = 51.5 ± 8.9 months CG: n = 32 (17F/15M); MA = 54.3 ± 7.9; PD = 53.2 ± 9.0 months	Unsupervised core exercises at home	Supervised in-person core exercises	Pain intensity (VAS) Functional disability (RMDQ and ODI)	Baseline, post (8 weeks), and 32 weeks	Intragroup SSI in VAS, RMDQ, and ODI (*p* < 0.001) Between-group SSI in favor of the CG in VAS post (*p* = 0.028), in RMDQ post (*p* = 0.004) and 32 weeks (*p* = 0.016), and in ODI post (*p* = 0.034)
Zadro, 2019 [42]	IG: n = 30 (18F/12M); MA = 68.8 ± 5.5; BMI = 26.9 ± 4.1 CG: n= 30 (13F/17M); MA = 67.8 ± 6.0; BMI = 27.4 ± 3.6	Unsupervised video game exercises at home	Usual activities of daily living	Pain intensity (NRS) Functional disability (PSFS and RMDQ)	Baseline and post (8 weeks)	Between-group SSI in favor of the IG in 11-point NRS (*p* = 0.04) and PSFS (*p* = 0.03), but not in RMDQ (*p* = 0.33)

BMI: body mass index (kg/m^2^); CG: control group; F: Female; FTST: five-times sit-to-stand test; IG: intervention group; M: Male; MA: mean age (years); NRS: 11-point numeric rating scale; ODI: Oswestry disability index; PD: pain duration; PSFS: patient-specific functional scale; RMDQ: Roland–Morris disability questionnaire; VAS: visual analogue scale. Data are presented as mean ± standard deviation. SSI: statistically significant improvement (*p*-value < 0.05).

**Table 2:** Detailed description of home interventions.

Author and Year	Type of Intervention	Frequency and Duration	Session Volume	Home Follow-Up	Aids, Tools, and Any Additional Aspects
Alp, 2014 [38]	CORE exercises (resistance)	6 weeks	1 set × 20 reps/exercise	Phone call 2 times/week	-
Dadarkhah, 2021 [39]	CORE exercises and lower limbs (resistance, stabilization, and mobility)	4 weeks 2 sessions/day 45 min/session	3 sets × 20–30 s/stretching 10–20 reps/exercise	Logbook Phone call 3 times/week	Illustrated booklet with exercises
Karaduman, 2023 [32]	CORE exercises (resistance and stabilization)	4 weeks 3 sessions/week 20–30 min/session	20–30 reps/exercise	IG 2: Online via video conference, under the guidance of a physiotherapist CG: Phone call every week	Progression from one position to another was achieved by holding the abdominal bracing for 8 s and completing 10 repetitions in the current position. CG: Illustrated booklet with exercises
Kuukkanen, 2007 [40]	CORE exercises and lower limbs (resistance and mobility)	12 weeks 7 sessions/week	3–4 sets × 15–20 reps/exercise	-	Detailed written explanation + illustrated booklet Progression with weekly tests + supervision once a month
Matarán-Peñarrocha, 2020 [41]	CORE exercises and lower limbs (resistance, stabilization, and mobility)	8 weeks 3 sessions/week 30–35 min/session	3 sets × 10–15 reps/exercise (except planks 30 s/exercise)	Logbook	Previous evaluation and face-to-face session (1 h) for explanation and execution of exercises + illustrated booklet Individualized program (tolerance and availability)
Zadro, 2019 [42]	Mobility, full-body resistance, and aerobic exercises through video games	8 weeks 3 sessions/week 60 min/session	-	Logbook Phone call every 2 weeks	Initial session (1–2 h) for correct and safe use Participants modified the parameters of the program The exercises included video and audio instructions, in addition to providing feedback during their execution

**Table 3:** Assessment of methodological quality by the PEDro scale.

Author and Year	1	2	3	4	5	6	7	8	9	10	11	Total	Quality
Alp, 2014 [38]	YES	YES	NO	YES	NO	NO	YES	YES	NO	YES	YES	6	High
Dadarkhah, 2021 [39]	YES	YES	NO	YES	NO	NO	NO	YES	NO	YES	YES	5	Moderate
Karaduma, 2023 [32]	YES	YES	NO	YES	NO	NO	YES	YES	NO	YES	YES	6	High
Kuukkanen, 2007 [40]	YES	YES	NO	YES	NO	NO	NO	YES	NO	YES	YES	5	Moderate
Matarán-Peñarrocha, 2020 [41]	YES	YES	YES	YES	NO	NO	YES	YES	YES	YES	YES	8	High
Zadro, 2019 [42]	YES	YES	YES	YES	NO	NO	YES	YES	YES	YES	YES	8	High

NO: the study does not present the criterion studied; YES: the study presents the criterion studied; 1: eligibility criteria were specified (this item is not taken into account for the final score); 2: subjects were randomly allocated to groups; 3: allocation was concealed; 4: the groups were similar at baseline regarding the most important prognostic indicators; 5: there was blinding of all subjects; 6: there was blinding of all therapists who administered the therapy; 7: there was blinding of all assessors who measured at least one key outcome; 8: measures of at least one key outcome were obtained from more than 85% of the subjects initially allocated to groups; 9: all subjects for whom outcome measures were available received the treatment or control condition as allocated or, where this was not the case, data for at least one key outcome was analyzed by “intention to treat”; 10: the results of between-group statistical comparisons are reported for at least one key outcome; 11: the study provides both point measures and measures of variability for at least one key outcome.

**Table 4:** Final meta-analytic model.

	Coefficient (SE)	95% CI	Z	^a^*p* Value	I^2^ (%)	R^2^
**Outcome effects**						
Functional disability	12.740 (SE = 3.791)	5.311, 20.170	3.361	0.001 *	68.392	1
Pain intensity	13.113 (SE = 3.987)	5.298, 20.928	3.289	0.001 *	80.710	0
**Covariable effects**						
Age over functional disability	−0.035 (SE = 0.050)	−0.133, 0.064	−0.687	0.492	56.882	
Age over pain intensity	−0.038 (SE = 0.053)	−0.141, 0.065	−0.725	0.468	72.930	
BMI over functional disability	−0.373 (SE = 0.123)	−0.615, −0.131	−3.021	0.003 *	2.492	
BMI over pain intensity	−0.375 (SE = 0.126)	−0.621, −0.129	−2.984	0.003 *	72.256	

BMI: body mass index (kg/m^2^); SE: standard error; 95% CI: 95% confidence interval. ^a^ significant if *p* < 0.05 (represented with *).

## Data Availability

The datasets generated during and/or analyzed during the current study are available from the corresponding author on reasonable request.

## Supplementary Materials

The following supporting information can be downloaded at: https://www.mdpi.com/article/10.3390/healthcare13172094/s1, Figure S1: Galaxy plot for combined outcomes; Figure S2: Caterpillar plot of measurements and studies; Figure S3: Funnel plot of measurements and studies (Numbers: number of results per study); Table S1: Search strategies, filters applied, and results obtained.

## Abbreviations

The following abbreviations are used in this manuscript:

AIC	Akaike information coefficient
BMI	Body mass index
FTSTS	Five-times sit-to-stand test
LBP	Low back pain
LRT	Likelihood ratio test
MD	Mean difference
MMA	Multivariate meta-analysis
NRS	Numeric rating scale
NSCLBP	Non-specific chronic low back pain
ODI	Oswestry disability index
PRISMA	Preferred reporting items for systematic reviews and meta-analyses
PSFS	Patient-specific functional scale
RCT	Randomized controlled trial
RMDQ	Roland–Morris disability questionnaire
SMD	Standardized mean difference
VAS	Visual analogue scale
