# Supplementary material for: Home Physical Exercise Interventions in Chronic Non-Specific Low Back Pain: Systematic Review and Multivariate Meta-Analysis
Source: Healthcare (Basel). 2025 Aug 22;13(17):2094. doi: 10.3390/healthcare13172094 (PMC12428419; doi:10.3390/healthcare13172094)

Supplementary Material

Table S1. Search strategies, filters applied and results obtained.

Search strategy	Database	Filters
(("Chronic low back pain"[Title/Abstract] OR "CLBP"[Title/Abstract] OR "Nonspecific chronic low back pain"[Title/Abstract] OR "Non-specific chronic low back pain"[Title/Abstract] OR "NSCLBP"[Title/Abstract] OR "NSLBP"[Title/Abstract] OR "Chronic nonspecific low back pain"[Title/Abstract] OR "Chronic non-specific low back pain"[Title/Abstract] OR "Chronic, nonspecific low back pain"[Title/Abstract] OR "Chronic, non-specific low back pain"[Title/Abstract]) AND ("Exercise*"[Title/Abstract] OR "Physical activit*"[Title/Abstract] OR "Physical exercise"[Title/Abstract] OR "Exercise training"[Title/Abstract] OR "Exercise therapy"[Title/Abstract])) AND ("Home environment"[Title/Abstract] OR "Home"[Title/Abstract] OR "Domiciliary"[Title/Abstract] OR "Remote"[Title/Abstract] OR "Video"[Title/Abstract] OR "Virtual"[Title/Abstract] OR "Telerehabilitation"[Title/Abstract] OR "Telemedicine"[Title/Abstract] OR "Mobile Health"[Title/Abstract] OR "Telehealth"[Title/Abstract] OR "mHealth"[Title/Abstract] OR "eHealth"[Title/Abstract])	PubMed	Title and abstract
((TS=("Chronic low back pain" OR "CLBP" OR "Nonspecific chronic low back pain" OR "Non-specific chronic low back pain" OR "NSCLBP" OR "NSLBP" OR "Chronic nonspecific low back pain" OR "Chronic non-specific low back pain" OR "Chronic, nonspecific low back pain" OR "Chronic, non-specific low back pain")) AND TS=("Exercise*" OR "Physical activit*" OR "Physical exercise" OR "Exercise training" OR "Exercise therapy")) AND TS=("Home environment" OR "Home" OR "Domiciliary" OR "Remote" OR "Video" OR "Virtual" OR "Telerehabilitation" OR "Telemedicine" OR "Mobile Health" OR "Telehealth" OR "mHealth" OR "eHealth"))	Web of Science	Title, abstract and keywords
(TITLE-ABS-KEY ("Chronic low back pain" OR "CLBP" OR "Nonspecific chronic low back pain" OR "Non-specific chronic low back pain" OR "NSCLBP" OR "NSLBP" OR "Chronic nonspecific low back pain" OR "Chronic non-specific low back pain" OR "Chronic, nonspecific low back pain" OR "Chronic, non-specific low back pain") AND TITLE-ABS-KEY ("Exercise*" OR "Physical activit*" OR "Physical exercise" OR "Exercise training" OR "Exercise therapy") AND TITLE-ABS-KEY ("Home environment" OR "Home" OR "Domiciliary" OR "Remote" OR "Video" OR "Virtual" OR "Telerehabilitation" OR "Telemedicine" OR "Mobile Health" OR "Telehealth" OR "mHealth" OR "eHealth"))	Scopus	Title, abstract and keywords
AB ("Chronic low back pain" OR "CLBP" OR "Nonspecific chronic low back pain" OR "Non-specific chronic low back pain" OR "NSCLBP" OR "NSLBP" OR "Chronic nonspecific low back pain" OR "Chronic non-specific low back pain" OR "Chronic, nonspecific low back pain" OR "Chronic, non-specific low back pain") AND AB ("Exercise*" OR "Physical activit*" OR "Physical exercise" OR "Exercise training" OR "Exercise therapy") AND AB ("Home environment" OR "Home" OR "Domiciliary" OR "Remote" OR "Video" OR "Virtual" OR "Telerehabilitation" OR "Telemedicine" OR "Mobile Health" OR "Telehealth" OR "mHealth" OR "eHealth")	SportDiscus	Abstract

Figure S1. Galaxy plot for combined outcomes.

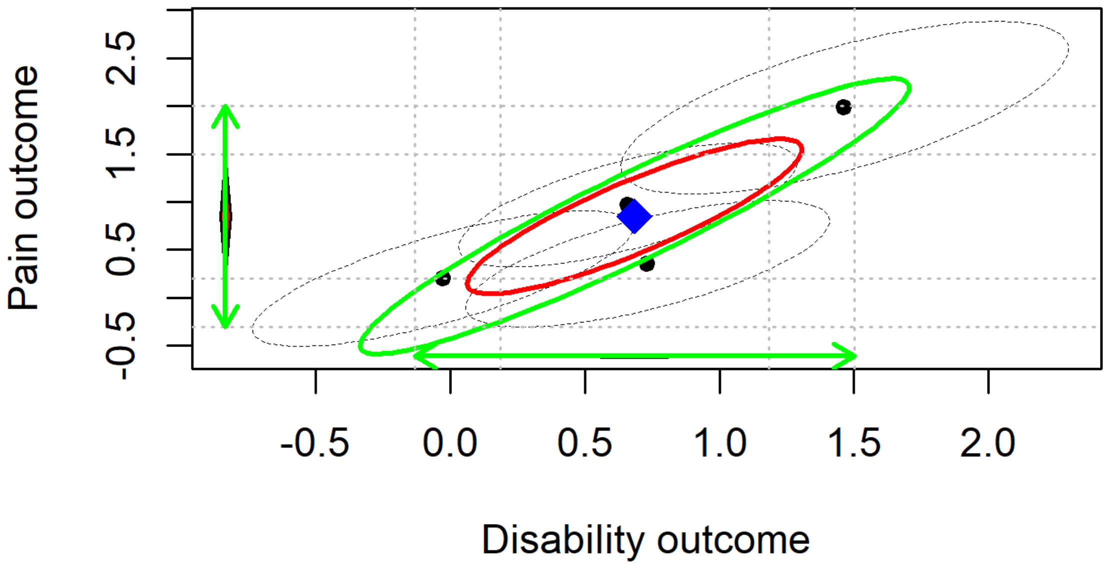

Figure S2. Caterpillar plot of measurements and studies.

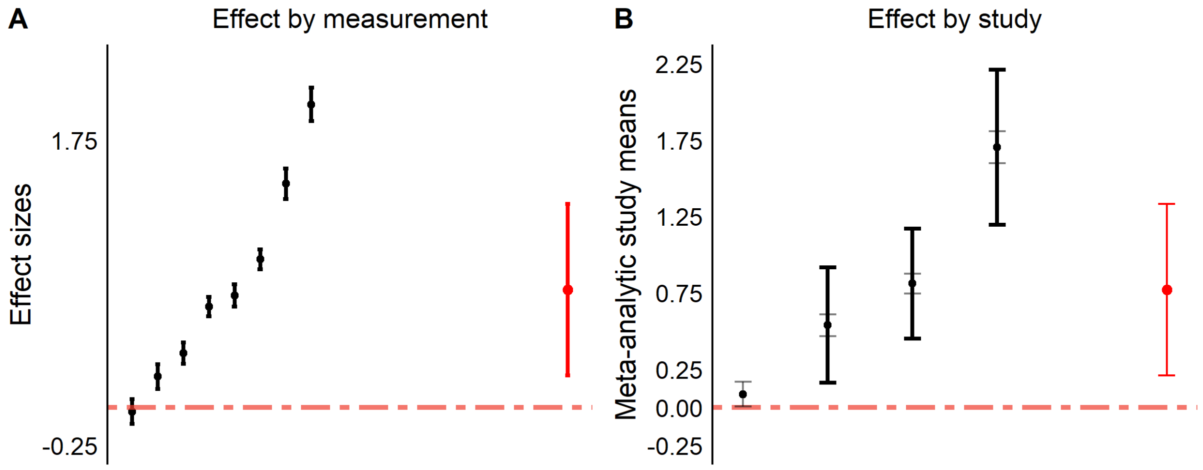

Figure S3. Funnel plot of measurements and studies (Numbers: number of results per study).

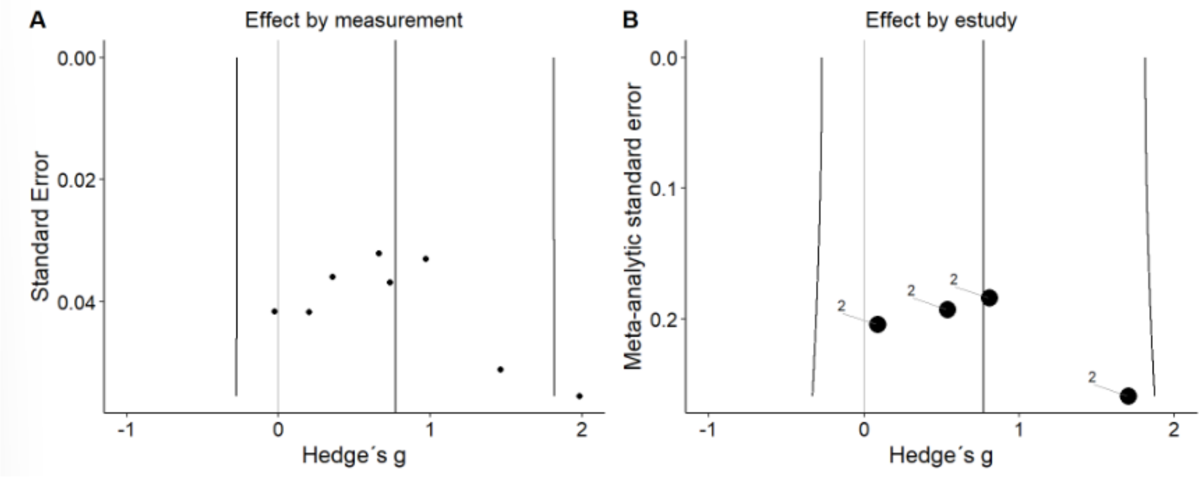

Supplement: Supplementary file 1 [file healthcare-13-02094-s001.zip › healthcare-3798857-supplementary.pdf]
